# NEAT1/MALAT1/XIST/PKD--Hsa-Mir-101-3p--DLGAP5 Axis as a Novel Diagnostic and Prognostic Biomarker Associated With Immune Cell Infiltration in Bladder Cancer

**DOI:** 10.3389/fgene.2022.892535

**Published:** 2022-07-08

**Authors:** Xiaosheng Rao, Haiyan Cao, Qingfeng Yu, Xiuyu Ou, Ruiqi Deng, Jinkun Huang

**Affiliations:** ^1^ Department of Urology, The First Affiliated Hospital of Guangzhou Medical University, Guangzhou, China; ^2^ Department of Nephrology, Tianjin Medical University General Hospital, Tianjin, China

**Keywords:** bladder cancer, DLGAP5, immune cell infiltration, RNA regulatory pathways, ceRNA network

## Abstract

**Background:** The clinical value of the biomarkers of bladder cancer (BC) is limited due to their low sensitivity or specificity. As a biomarker, DLG associated protein 5 (DLGAP5) is a potential cell cycle regulator in cancer cell carcinogenesis. However, its functional part in BC remains unclear. Therefore, this study aims to identify DLGAP5 expression in BC and its potential diagnostic and prognostic values. Eventually, it predicts the possible RNA regulatory pathways of BC.

**Methods:** Data on DLGAP5 expression levels in BC and normal bladder tissues were obtained from The Cancer Genome Atlas (TCGA) and the Gene Expression Omnibus (GEO) databases. The receiver operating characteristic (ROC), Kaplan–Meier survival curves, and the univariate and multivariate Cox regression analysis determined the diagnostic and prognostic values of DLGAP5 in BC patients. Finally, the StarBase predicted the target RNAs and constructed networks using Cytoscape.

**Results:** DLGAP5 expression was significantly upregulated in BC tissue, verified by the TCGA (*p* < 0.001), GSE3167, GSE7476, and GSE65635 datasets (*p* < 0.01). BC patients with increased DLGAP5 had poor overall survival (OS) (*p* = 0.01), disease specific survival (DSS) (*p* = 0.006) and progress free interval (DFI) (*p* = 0.007). The area under the ROC curve (AUC) was 0.913. The multivariate Cox analysis identified that lymphovascular invasion (*p* = 0.007) and DLGAP5 (*p* = 0.002) were independent prognostic factors.

**Conclusion:** Increased DLGAP5 expression was closely associated with a poor prognosis in BC patients. In this case, DLGAP5 might be a diagnostic and prognostic biomarker for BC. DLGAP5 expression might be regulated by NEAT1/MALAT1/XIST/PKD--Hsa-mir-101-3p pathways.

## Introduction

Bladder cancer (BC) is one of the most common urinary tumors globally and the 10th most common cancer. Around 549,393 new cases were diagnosed and 199,922 deaths were caused by BC worldwide in 2018 (Bray et al., 2018). Indeed, BC has significantly impacted patients’ health and brought a substantial economic burden (Antoni et al., 2017). Although there are various diagnostic methods for BC, it is prone to recurrence (Patel et al., 2020), and postoperative diagnosis typically requires invasive examination, known as cystoscopy (DeGeorge et al., 2017). Therefore, it is significant to look for new and non-invasive diagnostic markers. Suppose we can look for diagnostic and prognostic markers of BC, it is beneficial to the early diagnosis and better treatment to improve the prognosis of patients and reduce economic burden. Existing articles have reported that ubiquitin-conjugating enzyme E2C (UBE2C) (Morikawa et al., 2013), ubiquitin-like with PHD and ring-finger domains 1 (UHRF1), nuclear matrix protein22 (NMP-22), human complement factor H-related protein (Unoki et al., 2009), leucine-rich and immunoglobulin-like domains (LRIGs), and asymptomatic microhematuria (AMH) (Soria et al., 2019) are potential prognostic or diagnostic markers of BC. However, the molecular mechanisms of BC remain unclear. Furthermore, the clinical value of these markers is limited due to their low sensitivity or specificity (Ng et al., 2021; Tran et al., 2021). Hence, it is urgent to discover new markers of BC for early diagnosis and systematic treatment.

DLG associated protein 5 (DLGAP5), also called Human hepatoma up-regulated protein (HURP), has an important biological function as a signaling molecule for having a guanylate kinase-associated protein homology domain (Tong et al., 2014). Besides, DLGAP5 is a potential cell cycle regulator in cancer cell carcinogenesis (Horning et al., 2018). A previous study report that DLGAP5 is a mitotic regulator of tumorigenesis primarily in cell cycle progression, capture of kinetochores, and generation of proper tension across sister kinetochores. DLGAP5 can promote microtubule polymerization, bipolar spindle formation and the stability of microtubules (Wong and Fang, 2006). Another study find that DLGAP5 plays an important role in multicomponent complex which affect the growth or stability of spindle (Koffa et al., 2006). Eissa et al. (2014). found that the expression of DLGAP5 (HURP) RNA in urine sampler could be detected, which might be a biomarker of bilharzial and nonbilharzial bladder cancer. It is reported that DLGAP5 may be a potential prognosis factor in lung cancer, breast cancer, pancreatic cancer, endometrial cancer, and colorectal cancer (Shi et al., 2017; Branchi et al., 2019; Ke et al., 2020; Xu et al., 2020; Zhang et al., 2020). Patients with increased DLGAP5 expression have shown worse prognoses in above cancers.

Recently, bioinformatics analysis have been widely used in cancers to identify new biomarkers for improving diagnosis and treatment (Kristensen et al., 2014). Moreover, the ceRNA hypothesis was proposed by Salmena et al. (2011). In this paper, it described clearly the theory of ceRNA, which revealed a new interaction mechanism between RNA having a great biological significance. The ceRNA hypothesis may explain disease processes and provide opportunities for new therapies. For instance, it is known that ceRNA with the binding site of miRNA can combine with miRNA and keep it silent. (Thomson et al., 2016). As far as we can retrieve, this study is the first to analyze the differential expression and determine the diagnostic and prognostic values of DLGAP5 in BC. Eventually, it has explored the potential DLGAP5-related RNA regulatory pathways of BC.

## Material and Methods

### RNA-Sequencing Data and Bioinformatics Analysis

We obtained 10,534 samples from TCGA to conduct a pan-cancer analysis, including 33 types of tumors. Nineteen pan-carcinoma tissues and 414 tumor tissues of the bladder from TCGA were identified to analyze differential DLGAP5 expression. Additionally, the GSE3167, GSE7476, and GSE65635 datasets were obtained to validate the DLGAP5 expression level between normal and tumor tissues of the bladder from the GEO database.

### Diagnostic and Prognostic Analyses of DLGAP5

The Receiver Operating Characteristic (ROC) survival curve was constructed to analyze the diagnostic values of DLGAP5 in BC patients. Firstly, The Kaplan–Meier (KM) method constructed the overall survival (OS) curve, the disease specific survival (DSS) curve, and the progress-free interval (PFI) curve, illustrating the prognostic association between DLGAP5 expression level and OS, DSS, and PFI. Secondly, the univariate and multivariate Cox regression analysis analyzed the prognostic values of DLGAP5 in BC patients; when *p* < 0.05, the Cox regression analysis result is statistically significant.

### Construction of the Clinical Characteristics Table

The characteristic clinical information of 414 BC patients, including age, TNM stage, pathologic stage, histologic grade, lymphovascular invasion, whether a smoker or not, OS event, DSS event, and PFI event, were obtained from the TCGA database. Based on the DLGAP5 expression level, the DLGAP5-positive BC patients were divided into high and low expression groups. Then, the correlation between DLGAP5 expression level and clinical characteristics was analyzed.

### Construction of the Protein-Protein Interaction Network

Related genes of DLGAP5 were acquired to construct the PPI network using the STRING online tool (https://string-db.org/). The screening criteria were as follows: 1) minimum required interaction score: highest confidence (0.900); 2) maximum number of interactors to show no more than ten interactors.

### Enrichment Analysis

The R software (version 4.1.1) clusterProfiler package analyzed the GO enrichment of DLGAP5 and its related genes. A bubble plot was constructed for visualizing these enrichment results. The R software also performed the KEGG pathway enrichment analysis of DLGAP5 and its associated genes. When the adjusted *p*-value < 0.05, the enriched functions and pathways were identified as significant results.

### Association Analysis Between Immune Cell Infiltration and DLGAP5

The GSVA package with single-sample Gene Set Enrichment Analysis (ssGSEA) of the R software analyzed and visualized the association between DLGAP5 expression and 24 kinds of immune cells, including aDC, B cells, CD8 T cells, Cytotoxic cells, DC, Eosinophils, iDC, Macrophages, Mast cells, Neutrophils, NK CD56bright cells, NK CD56dim cells, NK cells, pDC, T cells, T helper cells, Tcm, Tem, TFH, Tgd, Th1 cells, Th17 cells, Th2 cells, and Treg. The applied statistical method was Spearman’s analysis. A lollipop graph was constructed to depict it. Then, the correlation between DLGAP5 expression and the enrichment of statistical immune cells was determined in more detail. For further exploration, the association between statistical immune cells and high/low-DLGAP5 was analyzed.

### Prediction of Target miRNAs of DLGAP5

It is reported that miRNAs can repress the function of mRNAs having a negative relation (McGeary et al., 2019). In our study, we want to explore the miRNAs of DLGAP5. The StarBase (version 2.0) (http://starbase.sysu.edu.cn/index.php) is a free and multifunctional database, which can provide the most comprehensive CLIP-seq data for exploring the interaction of all kinds of RNAs (Li et al., 2014). The StarBase can predict target miRNAs of DLGAP5 for us, including seven online miRNA databases, namely: PITA, RNA22, miRmap, microT, miRanda, PicTar, and TargetScan. The screening criteria were as follows: mammal, human, hg19, miRNA: all, CLIP-Data: high stringency (≥3), Degradome Data: with or without data, pan-cancer: with or without data. The target miRNAs were those found in at least three databases. Then, a correlation network of DLGAP5 and seven target miRNAs was constructed using the Cytoscape software.

### Construction of ceRNA Networks

The StarBase was also used to predict lncRNAs and circRNAs of seven selected target miRNAs. The screening criteria of lncRNA were as follows: mammal, human, hg19, CLIP Data: stringency> = 5, Degradome data: with and without data. The screening criteria of circRNA were as follows: mammal, human, hg19, CLIP Data: stringency > = 5, Degradome data: stringency > = 3. A ceRNA network was constructed encompassing all selected target miRNAs, lncRNAs, and circRNAs and DLGAP5 using the Cytoscape software.

### Prognostic Analysis of Hsa-Mir-124-3p and Hsa-Mir-101-3p

The pan-cancer analysis module of StarBase finished the survival analysis of hsa-mir-124-3p and hsa-mir-101-3p and the analysis of miRNA-DLGAP5 co-expression.

### Statistics Analysis

The R software was used to perform statistical analysis and visualization. The Chi-square test, the Fisher’s exact test, and the Wilcoxon rank-sum test were used in clinical information analysis (Wang et al., 2021). The Spearman’s analysis was used in immune cell infiltration. The cox regression analysis was used in the prognostic analysis of Kaplan–Meier. The Wilcoxon rank-sum test and matched *t*-test were used in the differential expression analysis. Log-rank test was used in the prognostic analysis of Hsa-mir-101-3p. Statistical significance was set at *p*-value <0.05 (Ge et al., 2021).

## Results

### Clinical Characteristics of BC Patients

The results determined that histologic grade (*p* < 0.001), lymphovascular invasion (*p* = 0.027), OS event (*p* = 0.046), DSS event (*p* = 0.023), and PFI event (*p* = 0.036) exhibited a statistical difference. No statistical difference in age (*p* = 0.618) and TNM stage (*p* = 0.721, 0.147, 0.882) was observed (Table 1).

**TABLE 1:** The clinical characteristics in BC with low and high expression of DLGAP5

Characteristic	Low expression of DLGAP5	High expression of DLGAP5	P
n	204	204	
Age, n (%)			0.618
<=70	112 (27.5%)	118 (28.9%)	
>70	92 (22.5%)	86 (21.1%)	
T stage, n (%)			0.721
T1	2 (0.5%)	1 (0.3%)	
T2	60 (16%)	59 (15.8%)	
T3	96 (25.7%)	98 (26.2%)	
T4	33 (8.8%)	25 (6.7%)	
N stage, n (%)			0.147
N0	116 (31.7%)	121 (33.1%)	
N1	18 (4.9%)	28 (7.7%)	
N2	45 (12.3%)	30 (8.2%)	
N3	4 (1.1%)	4 (1.1%)	
M stage, n (%)			0.882
M0	103 (49.8%)	93 (44.9%)	
M1	5 (2.4%)	6 (2.9%)	
Pathologic Stage, n (%)			0.637
Stage I	2 (0.5%)	0 (0%)	
Stage II	66 (16.3%)	64 (15.8%)	
Stage III	67 (16.5%)	73 (18%)	
Stage IV	68 (16.7%)	66 (16.3%)	
Histologic Grade, n (%)			<0.001^a^
High Grade	184 (45.4%)	200 (49.4%)	
Low Grade	19 (4.7%)	2 (0.5%)	
Lymphovascular invasion, n (%)			0.027^a^
No	55 (19.6%)	75 (26.7%)	
Yes	85 (30.2%)	66 (23.5%)	
Smoker, n (%)			0.385
No	59 (14.9%)	50 (12.7%)	
Yes	139 (35.2%)	147 (37.2%)	
OS event, n (%)			0.046^a^
Alive	125 (30.6%)	104 (25.5%)	
Dead	79 (19.4%)	100 (24.5%)	
DSS event, n (%)			0.023^a^
Alive	149 (37.8%)	123 (31.2%)	
Dead	51 (12.9%)	71 (18%)	
PFI event, n (%)			0.036^a^
Alive	128 (31.4%)	106 (26%)	
Dead	76 (18.6%)	98 (24%)	

a *p* <0.05.

### Increased Expression of DLGAP5 in BC

**FIGURE 1:**
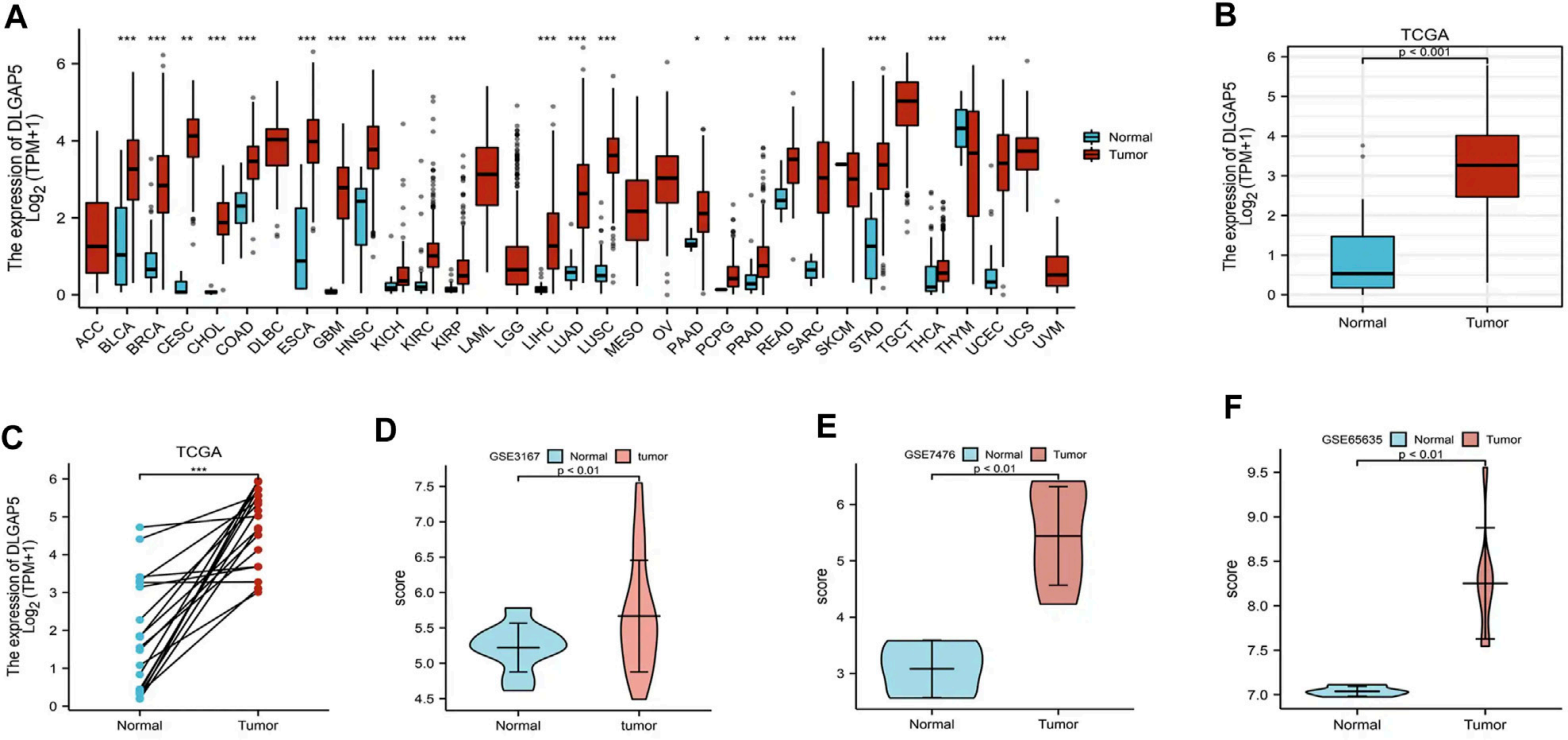
Different expression level of DLGAP5. **(A)** A graph was constructed to show the expression levels of DLGAP5 between normal tissue with bladder tumor tissue for 33 types of cancers. High expression of DLGAP5 in bladder tumor (***). **(B)** The different expression of DLGAP5 in bladder tumor tissues compared to adjacent tissues from TCGA database (p < 0.001). **(C)** The different expression of DLGAP5 in bladder tumor samples and matched adjacent samples from TCGA database (***). **(D)** GSE3167, **(E)** GSE7476 and **(F)** GSE65635 from GEO database were used to verify high expression of DLGAP5 in bladder tumor (p < 0.01). *: p < 0.05, **: p < 0.01, ***: p < 0.001.

A pan-cancer analysis of differential DLGAP5 expression was performed. It revealed that DLGAP5 expression level increased in BC, breast cancer, cervical squamous carcinoma, cholangiocarcinoma, colon adenocarcinoma, esophageal carcinoma, glioblastoma multiforme, head, and neck squamous cell carcinoma, kidney cancer, hepatocellular liver carcinoma, lung adenocarcinoma, lung squamous cell carcinoma, pancreatic adenocarcinoma, rectum adenocarcinoma, prostate adenocarcinoma, stomach adenocarcinoma, thyroid carcinoma, and uterine corpus endometrial carcinoma (Figure 1A). In addition, DLGAP5 expression level significantly increased in BC tissues than in normal tissues (*p* < 0.05), as validated from the TCGA database (*p* < 0.001) (Figures 1B,C) and the GSE3167, GSE7476, and GSE65635 datasets from the GEO database (*p* < 0.01) (Figures 1D–F).

### Diagnosis and Prognosis Analysis

**FIGURE 2:**
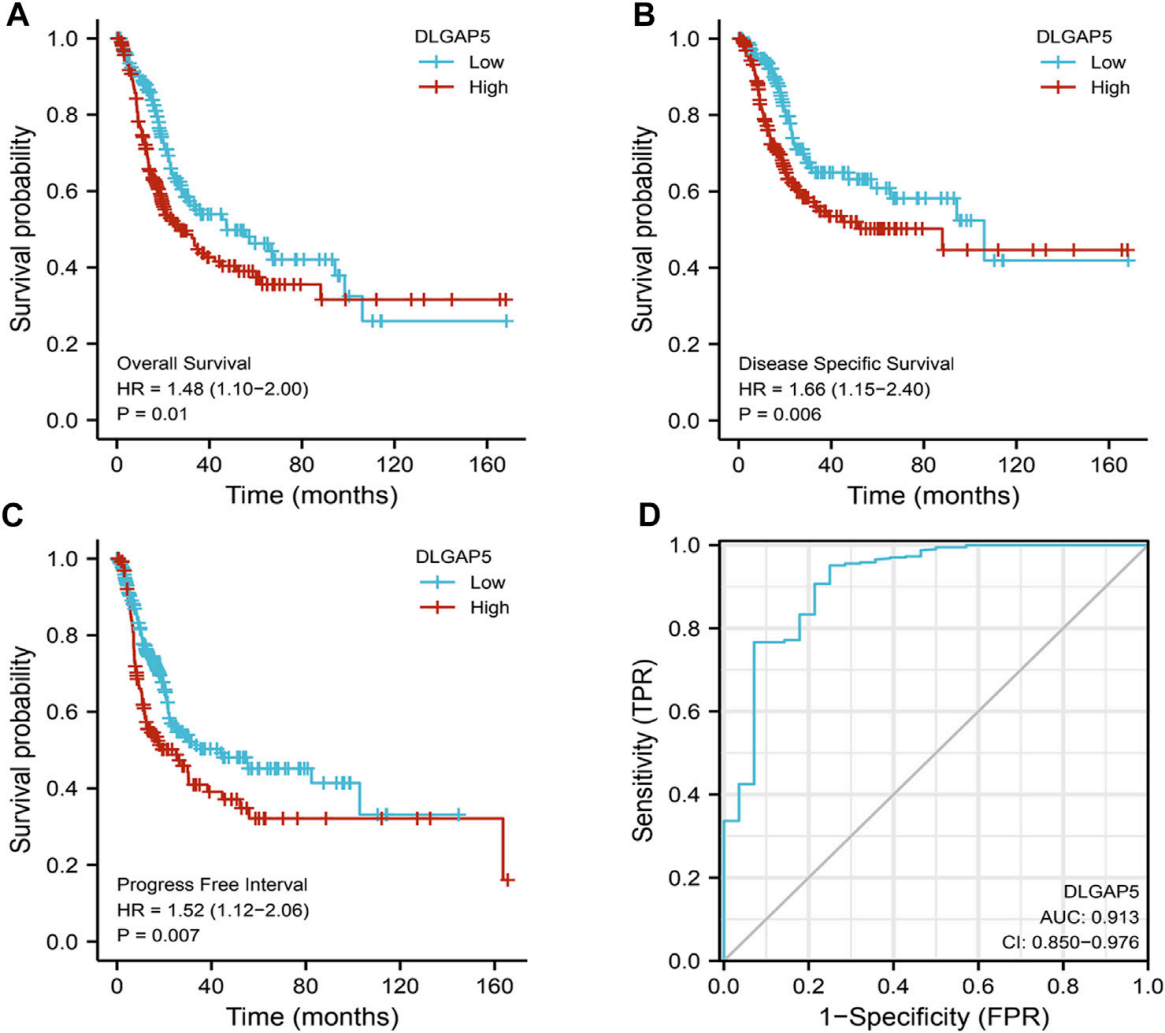
The diagnostic and prognostic analysis Of DLGAP5. **(A)** High DLGAP5 had worse overall survival (OS) for bladder cancer patients (p = 0.01, HR = 1.48). **(B)** The bladder cancer patients with high expression of DLGAP5 had worse disease specific survival (DSS) (p = 0.006, HR = 1.66). **(C)** The bladder cancer patients with high expression of DLGAP5 had worse progress free interval (DFI) (p = 0.007, HR-1.52). **(D)** The diagnostic analysis of DLGAP5 in bladder cancer patients. the area under the ROC curve (AUC) was 0.913, CI: 0.850–0.976. When the p < 0.05, the result was regarded as statistical significance.

The KM survival curves of OS (*p* = 0.01), DSS (*p* = 0.006), and PFI (*p* = 0.007) depicted that the increased DLGAP5 expression was associated with poor prognosis for BC patients (Figures 2A–C). The ROC survival curve was constructed to analyze the diagnostic values of DLGAP5 in BC patients. The result identified that the ROC curve (AUC) area was 0.913 (Figure 2D). The univariate Cox analysis revealed that age (*p* = 0.006), TNM stage (*p* < 0.001), pathologic stage (*p* < 0.001), lymphovascular invasion (*p* < 0.001), and DLGAP5 (*p* = 0.041) were associated with OS events. The multivariate Cox analysis determined that lymphovascular invasion (*p* = 0.007) and DLGAP5 (*p* = 0.002) were associated with OS events (Table 2).

**TABLE 2:** The univariate and multivariate cox analysis

Characteristics	Total (N)	Univariate analysis		Multivariate analysis	
		HR (95% CI)	P value	HR (95% CI)	P value
Age (< = 70 vs. > 70)	407	1.514 (1.128−2.031)	0.006^a^	1.165 (0.630−2.152)	0.627
Gender (Male vs. Female)	407	0.874 (0.631−1.210)	0.417		
T stage	373				
T1 and T2	122	Reference			
T stage (T1 + T2 vs. T3)	193	1.917 (1.302−2.824)	< 0.001^a^	3.155 (0.702−14.182)	0.134
T stage (T1 + T2 vs. T4)	58	2.867 (1.779−4.620)	< 0.001^a^	2.974 (0.616−14.354)	0.175
N stage (N0 + N1 vs. N2 + N3)	365	2.240 (1.609−3.120)	< 0.001^a^	1.459 (0.684−3.112)	0.329
M stage (M0 vs. M1)	207	3.310 (1.582−6.926)	0.001^a^	1.712 (0.456−6.425)	0.426
Pathologic stage (Stage I + Stage II vs. Stage III + Stage IV)	405	2.203 (1.521−3.190)	< 0.001^a^	0.416 (0.079−2.196)	0.301
Histologic grade (LG vs. HG)	404	2.899 (0.717−11.718)	0.135		
Lymphovascular invasion (No vs. Yes)	280	2.249 (1.547−3.268)	< 0.001^a^	2.696 (1.316−5.522)	0.007^a^
DLGAP5 (Low vs. High)	407	1.362 (1.013−1.831)	0.041^a^	2.925 (1.501−5.699)	0.002^a^

HR: Hazard ratio, LG: low grade, HG: High grade.

a *P* value < 0.05.

### PPI Network Analysis

**FIGURE 3:**
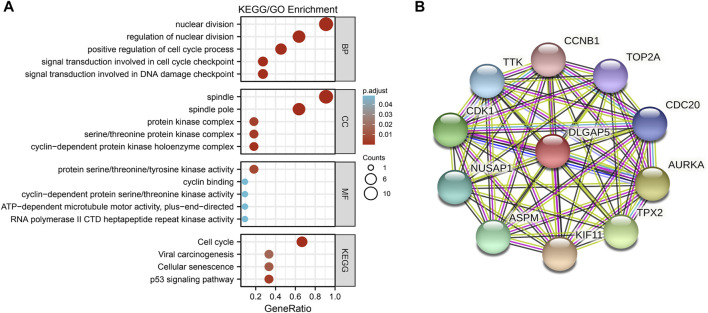
The GO/KEGG enrichment analysis and PPI network. **(A)** A bubble plot was used to show the results of the GO/KEGG enrichment. The color of bubble was on behalf of the adjusted value (p.adjust) and the size of bubble represent the degree of enrichment. **(B)** The PPI network was used to show DLGAP5 and its correlated genes including CCNB 1, TOP2A, CDC20, AURKA, TPX2, KIF11, ASPM, NUSAP1, CDK1, and TTK.

The PPI network included DLGAP5, CCNB1, TOP2A, CDC20, AURKA, TPX2, KIF11, ASPM, NUSAP1, CDK1, and TTK (Figure 3B).

### Enrichment Analysis

The GO enrichment included three parts: Molecular Function (MF), Biological Process (BP), and Cellular Component (CC). According to the adjusted *p*-value < 0.05 and q-value < 0.2, four KEGG pathways and five terms were selected per GO enrichment parts to perform a bubble plot (Figure 3A). The result indicated that KEGG pathways mainly focused on the cell cycle, p53 signaling pathway, cellular senescence, and viral carcinogenesis. The GO enrichment terms of BP were also mainly enriched in the nuclear division, the positive regulation of the cell cycle, the signal transduction involved in DNA damage checkpoint, the signal transduction involved in cell cycle checkpoint, and the regulation of nuclear division. The terms of CC included the spindle, the spindle pole, the serine/threonine protein kinase complex, the protein kinase complex, and the cyclin-dependent protein kinase holoenzyme complex. The terms of MF had protein serine/threonine/tyrosine kinase activity, cyclin-dependent protein serine/threonine kinase activity, ATP-dependent microtubule motor activity plus-end-directed, cyclin binding, RNA polymerase II and CTD heptapeptide repeat kinase activity. All selected pathways and terms are in Table 3.

**TABLE 3:** The overview of KEGG pathways and GO enrichment terms selected.

Ontology	ID	Description	p.adjust
BP	GO:0000280	Nuclear division	1.20042E-13
BP	GO:0090068	Positive regulation of cell cycle process	5.47663E-06
BP	GO:0072422	Signal transduction involved in DNA damage checkpoint	5.84843E-05
BP	GO:0072395	Signal transduction involved in cell cycle checkpoint	6.01927E-05
BP	GO:0051783	Regulation of nuclear division	2.18971E-10
CC	GO:0005819	Spindle	1.30276E-15
CC	GO:0000922	Spindle pole	1.86509E-11
CC	GO:1902554	Serine/threonine protein kinase complex	0.003376654
CC	GO:1902911	Protein kinase complex	0.00455199
CC	GO:0000307	Cyclin-dependent protein kinase holoenzyme complex	0.000825205
MF	GO:0004712	Protein serine/threonine/tyrosine kinase activity	0.006568804
MF	GO:0004693	Cyclin-dependent protein serine/threonine kinase activity	0.048549788
MF	GO:0008574	ATP-dependent microtubule motor activity, plus-end-directed	0.048549788
MF	GO:0030332	cyclin binding	0.048549788
MF	GO:0008353	RNA polymerase II CTD heptapeptide repeat kinase activity	0.048549788
KEGG	hsa04110	Cell cycle	5.45668E-06
KEGG	hsa04115	p53 signaling pathway	0.003542622
KEGG	hsa04218	Cellular senescence	0.012683523
KEGG	hsa05203	Viral carcinogenesis	0.016449973

KEGG: kyoto encyclopedia of genes and genomes, GO: gene ontology.

### Analysis of Immune Cell Infiltration With DLGAP5

**FIGURE 4:**
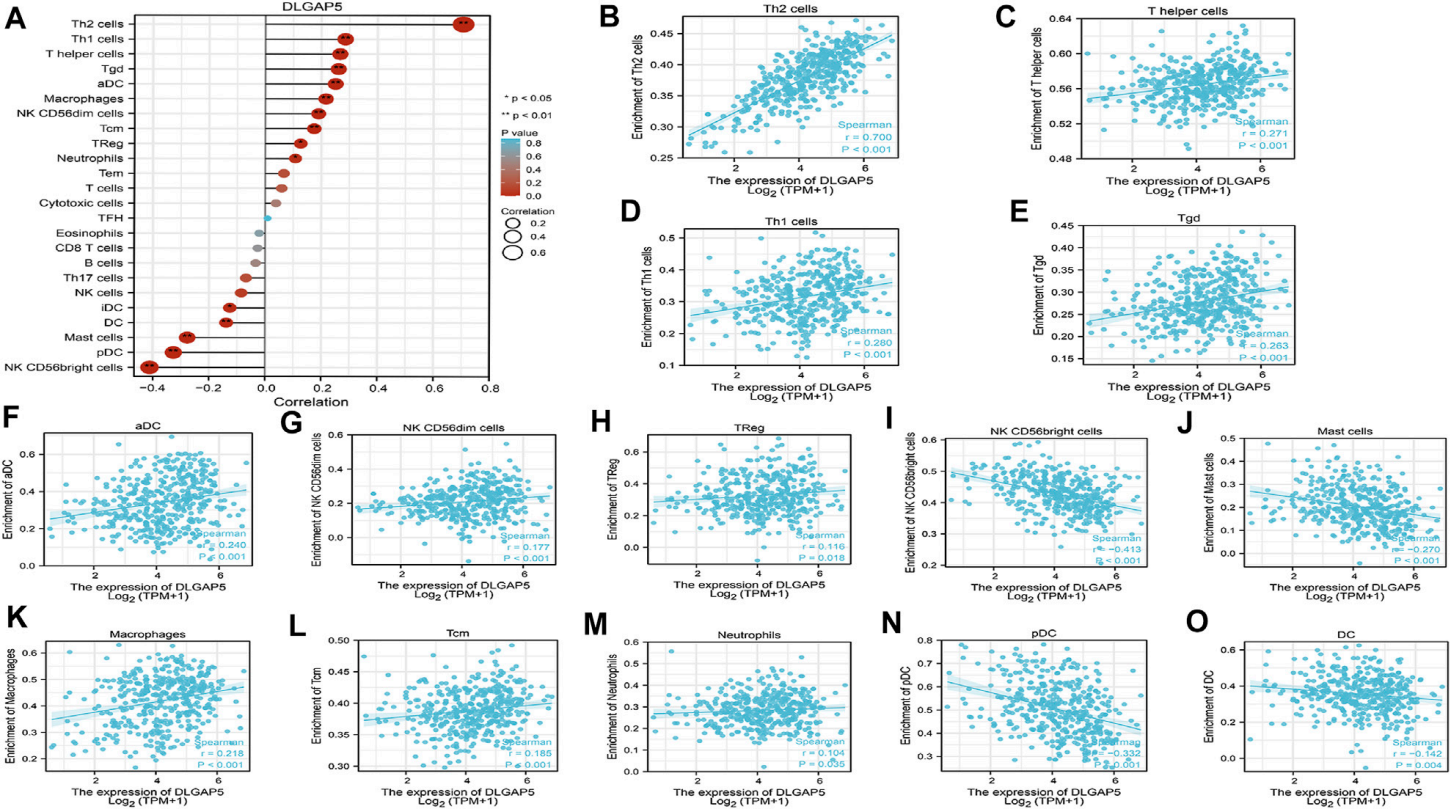
Analysis of immune cell infiltration with DLGAP5. **(A)** a lollipop graph was constructed to show the correlation between DLGAP5 with 24 types of immune cells. The different degree color of bubble was on behalf of the p value and the size of bubble represented the degree of correlation. The left terms were negative correlation and the right terms were positive correlation when p value < 0.05. 0: p < 0.05, a*: p < 0.01. **(B)** Th2 cells (p < 0.001,1-0.700) **(C)** T helper cells (p < 0.001, t-0.271) **(D)** Thl cells (p < 0.001,1-0.280) **(E)** Tgd (p < 0.001, r-0.263) **(F)** aDC (r < 0.001, r = 0.240) **(G)** NK CD56dim cells (p < 0.001, r = 0.177) **(H)** TReg (p = 1.018, r = 0.116) **(K)** Macrophages (p < 0.001, 1 = 0.218) **(L)** Tem (p < 0.01:11,-i = 0.185), and **(M)** Neutrophils (p-1.035, 1 = 0.104) had positive correlation with DLGAP5. **(I)** NK CD56bright cells (p < 0.001 **(N)** pDC (p < 0.001, r = −0.332) **(J)** Mast cells (p < 0.001, t = −0.270) and **(O)** DC (p = 0.004, r--0.142) had negative association with DLGAP5.

**FIGURE 5:**
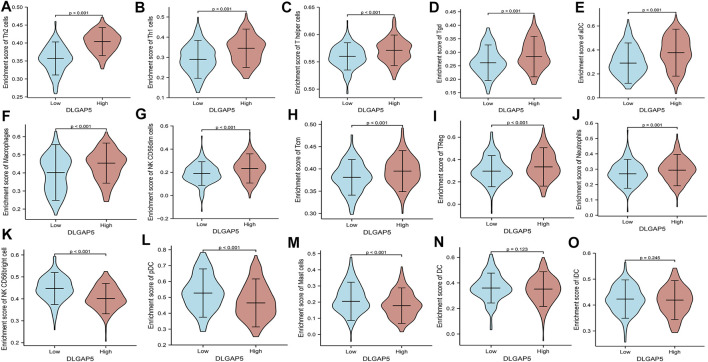
The enrichment of 15 kinds of statistical immune cells in high and low DLGAP5. Th2 cells (p < 0.001), Thl cells (p < 0.001), T helper cells (p < 0.001), Tgd (p < 0.001), aDC (p < 0.001), Macrophages (p < 0.001), NK CD56dim cells (p < 0.001), Tam (p < 0.001), TReg (p < 0.001) and Neutrophils (p = 0.001) had more higher enrichment level in high level than low level of DLGAP5 **(A–J)**, while NK CD56bright cells, pDC and Mast cells had more higher enrichment level in low level than high level of DLGAP5 **(K–M)**. DC (p,1).123 and iDC (p = 0.245) have no statistical significance between two groups **(N,O)**.

The R software was used to analyze and visualize the relationship between DLGAP5 and 24 kinds of immune cells and construct a lollipop graph (Figure 4A). The result identified that DLGAP5 expression level had a positive association with Th2 cells (*p* < 0.001, r = 0.700), Th1 cells (*p* < 0.001, r = 0.280), T helper cells (*p* < 0.001, r = 0.271), Tgd (*p* < 0.001, r = 0.263), aDC (*p* < 0.001, r = 0.240), Macrophages (*p* < 0.001, r = 0.218), NK CD56dim cells (*p* < 0.001, r = 0.177), Tcm (*p* < 0.001, r = 0.185), Treg (*p* = 0.018, r = 0.116), and Neutrophils (*p* = 0.035, r = 0.104), while NK CD56bright cells (*p* < 0.001, r = −0.413), pDC (*p* < 0.001, r = −0.332), Mast cells (*p* < 0.001, r = −0.270) and DC (*p* = 0.004, r = −0.142) had a negative association (Figure 4B–O). The enrichment of 15 kinds of statistical immune cells in high and low DLGAP5 were further analyzed; Th2 cells (*p* < 0.001), Th1 cells (*p* < 0.001), T helper cells (*p* < 0.001), Tgd (*p* < 0.001), aDC (*p* < 0.001), Macrophages (*p* < 0.001), NK CD56dim cells (*p* < 0.001), Tcm (*p* < 0.001), TReg (*p* < 0.001), and Neutrophils (*p* = 0.001) had higher enrichment in high DLGAP5 than in low DLGAP5 (Figures 5A–J), while NK CD56bright cells, pDC and Mast cells had higher enrichment in low DLGAP5 than in high DLGAP5 (Figures 5K–M). DC (*p* = 0.123) and iDC (*p* = 0.245) had no statistical significance between the two groups (Figures 5N,O).

### Prediction of Target miRNAs

**FIGURE 6:**
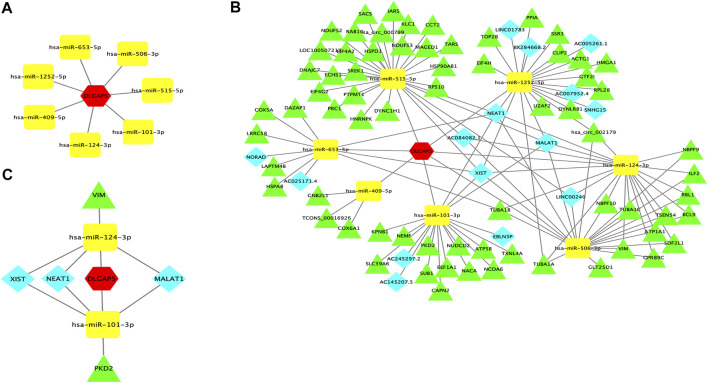
DLGAP5-miRNAs correlation network and ceRNA network. **(A)** A correlation network between DLGAP5 with seven target miRNAs. Seven target miRNAs of DLGAP5, including hsa-mir-101-3p, hsa-mir-124-3p, hsa-mir-409-5p, hsa-mir-515-5p, hsa-mir-506-3p, hsa-mir-653-5p, and hsa-mir-l252-5p, were predicted and selected by StarBase database. **(B)** DLGAP5 and all selected target miRNAs, lneRNAs and circRNAs were constructed a ceRNA network by using Cytoscape software. **(C)** The ceRNA network of the potential RNA regulatory pathways. The red hexagon represents DLGAP5, blue diamond represents lncRNA, yellow round rectangle represents miRNA, and green triangle represents circRNA.

The target miRNAs were selected in at least three databases. Seven target miRNAs were predicted by StarBase, including hsa-mir-101-3p, hsa-mir-124-3p, hsa-mir-409-5p, hsa-mir-515-5p, hsa-mir-506-3p, hsa-mir-653-5p, and hsa-mir-1252-5p. Cytoscape was used to construct a correlation network (Figure 6A).

### Prediction of Target LncRNAs and CircRNAs to Construct the ceRNA Network

StarBase was used to predict the lncRNAs and circRNAs of seven target miRNAs. A ceRNA network was constructed encompassing all selected target miRNAs, lncRNAs, and circRNAs, and DLGAP5 using Cytoscape software (Figure 6B). Finally, Hsa-mir-101-3p had 6 lncRNAs, and 12 circRNAs, Hsa-mir-124-3p had 5 lncRNAs, and 14 circRNAs, Hsa-mir-409-5p had 3 lncRNAs, and 3 circRNAs, Hsa-mir-515-5p had 2 lncRNAs, and 23 circRNAs, Hsa-mir-506-3p had 5 lncRNAs and 15 circRNAs, Hsa-mir-653-5p had 4 lncRNAs, and 6 circRNAs, and Hsa-mir-1252-5p had 8 lncRNAs, and 13 circRNAs. The ceRNA hypothesis indicated that miRNA was down-regulated when gene expression increased. We have retrieved papers in the PubMed database and selected downregulated miRNAs from seven target miRNAs; The selected miRNAs were hsa-mir-124-3p and hsa-miR-101-3p, which reported and confirmed deregulating expression in BC. Then, we selected three upregulated lncRNAs and two upregulated circRNAs of the two downregulated miRNAs in BC. The upregulated lncRNAs were NEAT1, MALAT1, and XIST, while the two upregulated circRNAs were PKD2, and VIM. Finally, a ceRNA network of potential RNA regulatory pathways to regulate BC was constructed (Figure 6C).

### Prognosis Analysis of Hsa-Mir-124-3p and Hsa-Mir-101-3p

**FIGURE 7:**
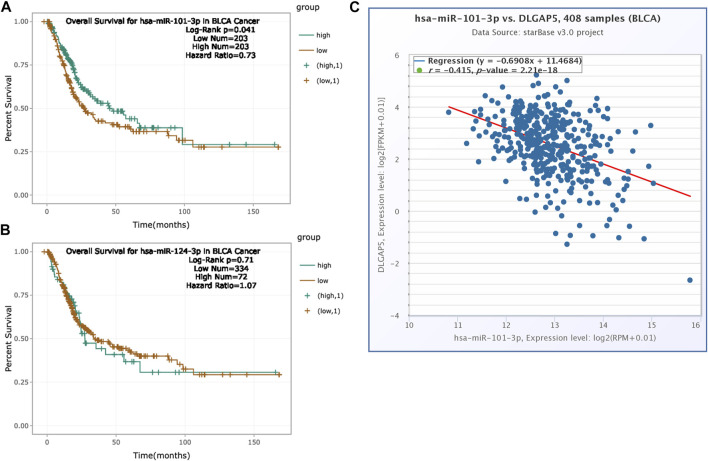
Prognosis analysis of hsa-mir-124-3p and hsa-mir-101-3p. **(A)** The K-M survival curve of hsa-miR-101-3p. High expression of hsa-miR-101-3p had positive overall survival for bladder cancer patients (Log-Rank p = 0.041, Hazard Ratio = 0.73). Green curve represented high expression of hsa-mir-101-3p and brown curve represented low expression of hsa-mir-101-3p. **(B)** The K-M survival curve of hsa-mir-124-3p. The result was no statistical significance (Log-Rank p = 0.71, Hazard Ratio = 1.07). **(C)** The co-expression between hsa-mir-101-3p with DLGAP5 in bladder cancer patients. The result shows that hsa-mir-101-3p expression level had negative relation with DLGAP5 expression level (r = −0.415).The p value was 2.21e-18.

The increased expression of hsa-mir-101-3p had a positive prognosis for BC patients (*p* = 0.041). The hazard ratio was 0.73 (Figure 7A). However, hsa-mir-124-3p had no significance for the prognosis of BC patients (*p* = 0.71) (Figure 7B). Hence, we explored the co-expression between hsa-mir-101-3p with DLGAP5 in BC. The result determined that the hsa-mir-101-3p expression level was negatively associated with DLGAP5 expression level (r = −0.415). The *p*-value was 2.21e-18 (Figure 7C), regarded as statistically significant.

## Discussion

The DLGAP5 expression profile in BC has not yet been systematically investigated. This study demonstrated that DLGAP5 was overexpressed and a negative prognostic factor for BC patients by bioinformatics analysis. Previous articles determined that DLGAP5 overexpression had also been shown in lung cancer, breast cancer, pancreatic cancer, endometrial cancer, and colorectal cancer (Shi et al., 2017; Branchi et al., 2019; Ke et al., 2020; Xu et al., 2020; Zhang et al., 2020). Meanwhile, increased DLGAP5 in the above cancers has an adverse prognosis. Coincidentally, the study’s findings were consistent with previous studies. Horning AM and colleagues demonstrated that DLGAP5 played an essential role in cancer cell carcinogenesis (Horning et al., 2018). The characteristic clinical analysis found that the expression of DLGAP5 had a significant relation with histologic grade. Therefore, this study hypothesizes that DLGAP5 plays a vital role in the pathogenesis of BC and may be an excellent prognosis biomarker for BC patients. The ROC analysis is typically used to estimate the diagnostic value, which has an excellent diagnostic efficiency (Mandrekar, 2010). As far as we can retrieve, DLGAP5 as a diagnostic marker of BC has not been reported. Surprisingly, we demonstrated that DLGAP5 had an excellent diagnostic performance in BC patients (AUC = 0.913), implying that DLGAP5 is a potential diagnostic biomarker of BC.

To clarify the biological role of DLGAP5 in BC, we investigated its most relative genes to perform GO/KEGG enrichment analysis. A previous study concluded that DLGAP5 was a mitotic spindle protein and targets cell cycle controllers (Horning et al., 2018). Furthermore, Hatfield et al. reported that DLGAP5 was a cell cycle gene associated with proliferation in acute myeloid leukemia patients (Hatfield et al., 2014). Meanwhile, Kuo and colleagues showed that DLGAP5 overexpression in HEK293 cells promoted the ubiquitination of p53 and its degradation by the proteasome (Kuo et al., 2012). Furthermore, after DLGAP5 overexpression, the expression of exogenous p53 in H1299 and Hep3b cells decreased, while their sensitivity to cisplatin-induced apoptosis also decreased. In this study, GO/KEGG enrichment analysis results demonstrated that DLGAP5 was a vital gene related to the cell cycle and p53 signaling pathways. Furthermore, DLGAP5 might be a novel therapeutic target of BC.

In this study, we demonstrated that DLGAP5 overexpression in BC patients was prone to lymphovascular invasion. Vittorio Branchi found that the knockdown of DLGAP5 led to a significant reduction of the invasion and migration in colorectal cancer (Branchi et al., 2019). Liao et al. (2013). reported that silencing the expression of DLGAP5 suppressed cell growth significantly and colony formation in hepatocellular carcinoma cells *in vitro*. Zhang et al. also reported that knockdown of DLGAP5 suppressed cell proliferation, induced G2/M phase arrest and apoptosis in ovarian cancer (Zhang et al., 2021). However, the functional experiment of DLGAP5 in BC was not observed. Based on the facts mentioned above, we hypothesized that DLGAP5 plays an essential role in tumorigenesis and the development of BC.

As we all know, the tumor microenvironment (TME) is complex and closely related to tumorigenesis and development, mainly composed of immune and stromal cells (Anderson and Simon, 2020). These cells can perform both tumor-promoting and anti-tumor functions. TME supports tumor growth and development, local invasion, metastasis, and spread (Pitt et al., 2016). Therefore, a viewpoint helped that TME was a new target for treating and preventing malignant tumors (Pitt et al., 2016; Zhou et al., 2019). In the present study, we found that the expression of DLGAP5 has the most positive correlation with Th2 cells and has the most negative correlation with NK CD56bright cells in BC. However, it may be beneficial to the further research of new therapeutic targets for BC patients.

The ceRNA hypothesis reveals a new interaction mechanism between RNA, which regulates the occurrence and development of diseases (Tay et al., 2014; Thomson et al., 2016; Yang et al., 2016). According to the ceRNA hypothesis, we selected two down-regulated miRNAs from predicted miRNAs, including hsa-mir-124-3p and hsa-mir-101-3p, reported and confirmed deregulating expression in BC (Zhang et al., 2014; Yuan et al., 2017; Li et al., 2019). Previous articles said that hsa-mir-101-3p was related to the proliferation and metastasis of BC cells (Liu et al., 2017; Li et al., 2019). Besides, previous studies showed that three lncRNAs, including NEAT1, MALAT1, and XIST (Hu et al., 2017; Guo et al., 2018; Jiao et al., 2018; Liu et al., 2019; Shan et al., 2020), and two circRNAs, including PKD2 and VIM (Fang et al., 2013; Li et al., 2018), were upregulated in BC. Furthermore, NEAT1, MALAT1, XIST, and PKD had reported or validated having a close relation with pro chemotherapy resistance of malignant tumors (Gutschner et al., 2013; Hu et al., 2017; Kim et al., 2018; Li et al., 2018; Shan et al., 2020). However, we demonstrated that high expression of hsa-mir-101-3p has an overall adverse survival for BC patients, which indicated that hsa-mir-101-3p might be a protective factor having a negative relation with DLGAP5 in BC patients. The above result was in accord with the ceRNA hypothesis. Eventually, we proposed the lncRNA‐miRNA‐mRNA pathway: NEAT1/MALAT1/XIST‐‐hsa‐mir‐101‐3p‐‐DLGAP5 and the circRNA‐miRNA‐mRNA pathway: PKD‐‐hsa‐mir‐101‐3p‐‐DLGAP5 are potential RNA regulatory pathways of BC, which regulated the occurrence, development, metastasis, chemosensitivity resistance of BC. There is no doubt that this study has some limitations. First, DLGAP5 requires validation in further fundamental studies. Some experiments will be conducted to verify the conclusions. Second, the number of normal samples is small.

## Conclusion

This study found that the increased expression of DLGAP5 had an unfavorable prognosis for BC patients and DLGAP5 might be a diagnostic and prognostic biomarker for BC. Additionally, NEAT1/MALAT1/XIST/PKD--hsa-mir-101-3p might regulate DLGAP5 expression, providing the basis for the further study of BC.

## Data Availability

The datasets presented in this study can be found in online repositories. The names of the repository/repositories and accession number(s) can be found in the article/Supplementary Material.

## References

[B1] Anderson N. M. Simon M. C. (2020). The Tumor Microenvironment. Curr. Biol. 30, R921–R925. 10.1016/j.cub.2020.06.081 32810447 PMC8194051

[B2] Antoni S. Ferlay J. Soerjomataram I. Znaor A. Jemal A. Bray F. (2017). Bladder Cancer Incidence and Mortality: A Global Overview and Recent Trends. Eur. Urol. 71, 96–108. 10.1016/j.eururo.2016.06.010 27370177

[B3] Branchi V. García S. A. Radhakrishnan P. Győrffy B. Hissa B. Schneider M. (2019). Prognostic Value of DLGAP5 in Colorectal Cancer. Int. J. Colorectal Dis. 34, 1455–1465. 10.1007/s00384-019-03339-6 31286215

[B4] Bray F. Ferlay J. Soerjomataram I. Siegel R. L. Torre L. A. Jemal A. (2018). Global Cancer Statistics 2018: GLOBOCAN Estimates of Incidence and Mortality Worldwide for 36 Cancers in 185 Countries. CA A Cancer J. Clin. 68, 394–424. 10.3322/caac.21492 30207593

[B5] DeGeorge K. C. Holt H. R. Hodges S. C. (2017). Bladder Cancer: Diagnosis and Treatment. Am. Fam. Physician 96, 507–514. 29094888

[B6] Eissa S. Matboli M. Mansour A. Mohamed S. Awad N. Kotb Y. M. (2014). Evaluation of Urinary HURP mRNA as a Marker for Detection of Bladder Cancer: Relation to Bilharziasis. Med. Oncol. 31 (2), 804. 10.1007/s12032-013-0804-4 24375315

[B7] Fang Z.-Q. Zang W.-D. Chen R. Ye B.-W. Wang X.-W. Yi S.-H. (2013). Gene Expression Profile and Enrichment Pathways in Different Stages of Bladder Cancer. Genet. Mol. Res. 12, 1479–1489. 10.4238/2013.May.6.1 23765955

[B8] Ge X. Chen Y. E. Song D. McDermott M. Woyshner K. Manousopoulou A. (2021). Clipper: P-value-free FDR Control on High-Throughput Data from Two Conditions. Genome Biol. 22 (1), 288. 10.1186/s13059-021-02506-9 34635147 PMC8504070

[B9] Guo Y. Zhang H. Xie D. Hu X. Song R. Zhu L. (2018). Non-coding RNA NEAT1/miR-214-3p Contribute to Doxorubicin Resistance of Urothelial Bladder Cancer Preliminary through the Wnt/β-Catenin Pathway. Cancer Manag. Res. 10, 4371–4380. 10.2147/CMAR.S171126 30349370 PMC6187925

[B10] Gutschner T. Hämmerle M. Eissmann M. Hsu J. Kim Y. Hung G. (2013). The Noncoding RNA MALAT1 Is a Critical Regulator of the Metastasis Phenotype of Lung Cancer Cells. Cancer Res. 73, 1180–1189. 10.1158/0008-5472.CAN-12-2850 23243023 PMC3589741

[B11] Hatfield K. J. Reikvam H. Bruserud Ø. (2014). Identification of a Subset of Patients with Acute Myeloid Leukemia Characterized by Long-Termin Vitroproliferation and Altered Cell Cycle Regulation of the Leukemic Cells. Expert Opin. Ther. Targets 18, 1237–1251. 10.1517/14728222.2014.957671 25200484

[B12] Horning A. M. Wang Y. Lin C.-K. Louie A. D. Jadhav R. R. Hung C.-N. (2018). Single-Cell RNA-Seq Reveals a Subpopulation of Prostate Cancer Cells with Enhanced Cell-Cycle-Related Transcription and Attenuated Androgen Response. Cancer Res. 78, 853–864. 10.1158/0008-5472.CAN-17-1924 29233929 PMC5983359

[B13] Hu Y. Deng C. Zhang H. Zhang J. Peng B. Hu C. (2017). Long Non-coding RNA XIST Promotes Cell Growth and Metastasis through Regulating miR-139-5p Mediated Wnt/β-Catenin Signaling Pathway in Bladder Cancer. Oncotarget 8, 94554–94568. 10.18632/oncotarget.21791 29212249 PMC5706895

[B14] Jiao D. Li Z. Zhu M. Wang Y. Wu G. Han X. (2018). LncRNA MALAT1 Promotes Tumor Growth and Metastasis by Targeting miR-124/foxq1 in Bladder Transitional Cell Carcinoma (BTCC). Am. J. Cancer Res. 8, 748–760. 29736319 PMC5934564

[B15] Ke M.-j. Ji L.-d. Li Y.-x. (2020). Bioinformatics Analysis Combined with Experiments to Explore Potential Prognostic Factors for Pancreatic Cancer. Cancer Cell. Int. 20, 382. 10.1186/s12935-020-01474-7 32782440 PMC7414559

[B16] Kim J. Piao H.-L. Kim B.-J. Yao F. Han Z. Wang Y. (2018). Long Noncoding RNA MALAT1 Suppresses Breast Cancer Metastasis. Nat. Genet. 50, 1705–1715. 10.1038/s41588-018-0252-3 30349115 PMC6265076

[B17] Koffa M. D. Casanova C. M. Santarella R. Köcher T. Wilm M. Mattaj I. W. (2006). HURP Is Part of a Ran-dependent Complex Involved in Spindle Formation. Curr. Biol. 16, 743–754. 10.1016/j.cub.2006.03.056 16631581

[B18] Kristensen V. N. Lingjærde O. C. Russnes H. G. Vollan H. K. M. Frigessi A. Børresen-Dale A.-L. (2014). Principles and Methods of Integrative Genomic Analyses in Cancer. Nat. Rev. Cancer 14, 299–313. 10.1038/nrc3721 24759209

[B19] Kuo T.-C. Chang P.-Y. Huang S.-F. Chou C.-K. Chao C. C.-K. (2012). Knockdown of HURP Inhibits the Proliferation of Hepacellular Carcinoma Cells via Downregulation of Gankyrin and Accumulation of P53. Biochem. Pharmacol. 83, 758–768. 10.1016/j.bcp.2011.12.034 22230478

[B20] Li B. Xie D. Zhang H. (2019). MicroRNA-101-3p Advances Cisplatin Sensitivity in Bladder Urothelial Carcinoma through Targeted Silencing EZH2. J. Cancer 10, 2628–2634. 10.7150/jca.33117 31258770 PMC6584933

[B21] Li J. H. Liu S. Zhou H. Qu L. H. Yang J. H. (2014). starBase v2.0: Decoding miRNA-ceRNA, miRNA-ncRNA and Protein-RNA Interaction Networks from Large-Scale CLIP-Seq Data. Nucleic Acids Res. 42 (Database issue), D92–D97. 10.1093/nar/gkt1248 24297251 PMC3964941

[B22] Li Q. Q. Hsu I. Sanford T. Railkar R. Balaji N. Sourbier C. (2018). Protein Kinase D Inhibitor CRT0066101 Suppresses Bladder Cancer Growth *In Vitro* and Xenografts via Blockade of the Cell Cycle at G2/M. Cell. Mol. Life Sci. 75, 939–963. 10.1007/s00018-017-2681-z 29071385 PMC7984729

[B23] Liao W. Liu W. Yuan Q. Liu X. Ou Y. He S. (2013). Silencing of DLGAP5 by siRNA Significantly Inhibits the Proliferation and Invasion of Hepatocellular Carcinoma Cells. PloS One 8, e80789. 10.1371/journal.pone.0080789 24324629 PMC3851768

[B24] Liu D. Li Y. Luo G. Xiao X. Tao D. Wu X. (2017). LncRNA SPRY4-IT1 Sponges miR-101-3p to Promote Proliferation and Metastasis of Bladder Cancer Cells through Up-Regulating EZH2. Cancer Lett. 388, 281–291. 10.1016/j.canlet.2016.12.005 27998761

[B25] Liu P. Li X. Cui Y. Chen J. Li C. Li Q. (2019). LncRNA-MALAT1 Mediates Cisplatin Resistance via miR-101-3p/VEGF-C Pathway in Bladder Cancer. Acta Biochim. Biophys. Sin. (Shanghai). 51, 1148–1157. 10.1093/abbs/gmz112 31650173

[B26] Mandrekar J. N. (2010). Receiver Operating Characteristic Curve in Diagnostic Test Assessment. J. Thorac. Oncol. 5, 1315–1316. 10.1097/JTO.0b013e3181ec173d 20736804

[B27] McGeary S. E. Lin K. S. Shi C. Y. Pham T. M. Bisaria N. Kelley G. M. (2019). The Biochemical Basis of microRNA Targeting Efficacy. Science 366 (6472), eaav1741. 10.1126/science.aav1741 31806698 PMC7051167

[B28] Morikawa T. Kawai T. Abe H. Kume H. Homma Y. Fukayama M. (2013). UBE2C Is a Marker of Unfavorable Prognosis in Bladder Cancer after Radical Cystectomy. Int. J. Clin. Exp. Pathol. 6, 1367–1374. 23826418 PMC3693202

[B29] Ng K. Stenzl A. Sharma A. Vasdev N. (2021). Urinary Biomarkers in Bladder Cancer: A Review of the Current Landscape and Future Directions. Urologic Oncol. Seminars Orig. Investigations 39, 41–51. 10.1016/j.urolonc.2020.08.016 32919875

[B30] Patel V. G. Oh W. K. Galsky M. D. (2020). Treatment of Muscle‐invasive and Advanced Bladder Cancer in 2020. CA A Cancer J. Clin. 70, 404–423. 10.3322/caac.21631 32767764

[B31] Pitt J. M. Marabelle A. Eggermont A. Soria J.-C. Kroemer G. Zitvogel L. (2016). Targeting the Tumor Microenvironment: Removing Obstruction to Anticancer Immune Responses and Immunotherapy. Ann. Oncol. 27, 1482–1492. 10.1093/annonc/mdw168 27069014

[B32] Salmena L. Poliseno L. Tay Y. Kats L. Pandolfi P. P. (2011). A ceRNA Hypothesis: the Rosetta Stone of a Hidden RNA Language? Cell. 146 (3), 353–358. 10.1016/j.cell.2011.07.014 21802130 PMC3235919

[B33] Shan G. Tang T. Xia Y. Qian H.-J. (2020). Long Non-coding RNA NEAT1 Promotes Bladder Progression through Regulating miR-410 Mediated HMGB1. Biomed. Pharmacother. 121, 109248. 10.1016/j.biopha.2019.109248 31734579

[B34] Shi Y.-X. Yin J.-Y. Shen Y. Zhang W. Zhou H.-H. Liu Z.-Q. (2017). Genome-scale Analysis Identifies NEK2, DLGAP5 and ECT2 as Promising Diagnostic and Prognostic Biomarkers in Human Lung Cancer. Sci. Rep. 7, 8072. 10.1038/s41598-017-08615-5 28808310 PMC5556079

[B35] Soria F. Krabbe L.-M. Todenhöfer T. Dobruch J. Mitra A. P. Inman B. A. (2019). Molecular Markers in Bladder Cancer. World J. Urol. 37, 31–40. 10.1007/s00345-018-2503-4 30259123 PMC6510866

[B36] Tay Y. Rinn J. Pandolfi P. P. (2014). The Multilayered Complexity of ceRNA Crosstalk and Competition. Nature 505, 344–352. 10.1038/nature12986 24429633 PMC4113481

[B37] Thomson D. W. Dinger M. E. (2016). Endogenous microRNA Sponges: Evidence and Controversy. Nat. Rev. Genet. 17, 272–283. 10.1038/nrg.2016.20 27040487

[B38] Tong J. Yang H. Eom S. H. Chun C. Im Y. J. (2014). Structure of the GH1 Domain of Guanylate Kinase-Associated Protein from *Rattus norvegicus* . Biochem. Biophysical Res. Commun. 452 (1), 130–135. 10.1016/j.bbrc.2014.08.073 25152391

[B39] Tran L. Xiao J.-F. Agarwal N. Duex J. E. Theodorescu D. (2021). Advances in Bladder Cancer Biology and Therapy. Nat. Rev. Cancer 21, 104–121. 10.1038/s41568-020-00313-1 33268841 PMC10112195

[B40] Unoki M. Kelly J. D. Neal D. E. Ponder B. A. J. Nakamura Y. Hamamoto R. (2009). UHRF1 Is a Novel Molecular Marker for Diagnosis and the Prognosis of Bladder Cancer. Br. J. Cancer 101, 98–105. 10.1038/sj.bjc.6605123 19491893 PMC2713709

[B41] Wang N. Lefaudeux D. Mazumder A. Li J. J. Hoffmann A. (2021). Identifying the Combinatorial Control of Signal-dependent Transcription Factors. PLoS Comput. Biol. 17 (6), e1009095. 10.1371/journal.pcbi.1009095 34166361 PMC8263068

[B42] Wong J. Fang G. (2006). HURP Controls Spindle Dynamics to Promote Proper Interkinetochore Tension and Efficient Kinetochore Capture. J. Cell. Biol. 173, 879–891. 10.1083/jcb.200511132 16769820 PMC2063914

[B43] Xu T. Dong M. Li H. Zhang R. Li X. (2020). Elevated mRNA Expression Levels of DLGAP5 Are Associated with Poor Prognosis in Breast Cancer. Oncol. Lett. 19, 4053–4065. 10.3892/ol.2020.11533 32391106 PMC7204629

[B44] Yang C. Wu D. Gao L. Liu X. Jin Y. Wang D. (2016). Competing Endogenous RNA Networks in Human Cancer: Hypothesis, Validation, and Perspectives. Oncotarget 7, 13479–13490. 10.18632/oncotarget.7266 26872371 PMC4924655

[B45] Yuan Q. Sun T. Ye F. Kong W. Jin H. (2017). MicroRNA-124-3p Affects Proliferation, Migration and Apoptosis of Bladder Cancer Cells through Targeting AURKA. Cancer Biomark. 19, 93–101. 10.3233/CBM-160427 28269755 PMC13020699

[B46] Zhang H. Liu Y. Tang S. Qin X. Li L. Zhou J. (2021). Knockdown of DLGAP5 Suppresses Cell Proliferation, Induces G2/M Phase Arrest and Apoptosis in Ovarian Cancer. Exp. Ther. Med. 22, 1245. 10.3892/etm.2021.10680 34539841 PMC8438692

[B47] Zhang T. Wang J. Zhai X. Li H. Li C. Chang J. (2014). MiR-124 Retards Bladder Cancer Growth by Directly Targeting CDK4. Acta Biochim. Biophys. Sin. (Shanghai). 46, 1072–1079. 10.1093/abbs/gmu105 25348738

[B48] Zhang W. Gao L. Wang C. Wang S. Sun D. Li X. (2020). Combining Bioinformatics and Experiments to Identify and Verify Key Genes with Prognostic Values in Endometrial Carcinoma. J. Cancer 11, 716–732. 10.7150/jca.35854 31942195 PMC6959041

[B49] Zhou F. Feng B. Yu H. Wang D. Wang T. Ma Y. (2019). Tumor Microenvironment‐Activatable Prodrug Vesicles for Nanoenabled Cancer Chemoimmunotherapy Combining Immunogenic Cell Death Induction and CD47 Blockade. Adv. Mat. 31, 1805888. 10.1002/adma.201805888 30762908

